# Family member involvement when multiple family members have type 1 diabetes

**DOI:** 10.1111/dme.70145

**Published:** 2025-10-06

**Authors:** Michelle L. Litchman, Cynthia A. Berg, Margot Porter, MaryJane S. Campbell, Eunjin Lee Tracy, Robert G. Kent de Grey, Chloe Mitchell, Nancy A. Allen

**Affiliations:** ^1^ University of Utah College of Nursing Salt Lake City Utah USA; ^2^ Department of Psychology University of Utah Salt Lake City Utah USA; ^3^ School of Medicine University of Utah Salt Lake City Utah USA

**Keywords:** chronic disease self‐management, family studies, psychosocial, qualitative methods, type 1 diabetes

## Abstract

**Aims:**

Family involvement is beneficial for type 1 diabetes (T1D) self‐management; however, few studies have examined family involvement when multiple family members (MFM) have T1D. This qualitative study examined the perceptions and experiences of family members (parents with and without diabetes, children with T1D, siblings without diabetes) where at least 2 members of the immediate family had T1D.

**Materials and Methods:**

Semi‐structured interviews were conducted with members from five families (*N* = 21).

**Results:**

Qualitative themes emerged noting the benefits of having MFM with T1D for initial diagnosis and the enhanced cohesion that comes from the support MFM with T1D provide each other. Across families, even when a father had T1D and the mother did not, the mother was described as the key source of support for MFM with T1D. This increased mental load and responsibilities of mothers come with risks to mothers' physical and emotional well‐being. Additionally, there were added family stressors, including enhanced financial burden, glucose levels interfering with family activities, and respecting differences among family members in management strategies. Finally, siblings without T1D noted frustrations and well‐being concerns due to children with T1D being prioritized over them.

**Conclusions:**

The findings add a rich view of family involvement in families where MFM experience T1D, an important contribution to a literature focused nearly exclusively on families where only one person has T1D. Family members with and without T1D are providing substantial support that is largely quite effective. The findings have clear clinical implications, especially regarding the needed support for mothers, for financial support and for siblings without diabetes.

1

**What's new?**

- Families with multiple members with T1D (MFM) experience unique benefits, including earlier diagnosis, improved self‐care through shared lived experience, and increased family cohesion—despite significant caregiving and financial burdens.
- Mothers in MFM families face disproportionate caregiving stress, including sleep disruption and emotional distress, underscoring the need for tailored, stepped‐care interventions and expanded support networks.
- Sibling involvement in diabetes care can lead to developmentally inappropriate roles, such as acting like a parent, suggesting a need for safeguards and education to ensure safe and supportive sibling engagement.

## 2 INTRODUCTION

Some 1.5 million Americans have T1D.^1^ Type 1 diabetes involves rigorous daily self‐care including monitoring blood glucose, insulin bolusing, adjusting insulin, as well as managing food and exercise.^2^ Family involvement from parents, siblings and romantic partners that typically do not have diabetes is beneficial.^3^ For example, when parents assist their children in a warm manner, children feel comfortable disclosing problems when they occur, which allows parents to be knowledgeable about diabetes management, resulting in better diabetes outcomes.^3^

Most research has examined family involvement in diabetes management in one family member (either a child or an adult) with T1D.^4^ Research typically excludes families from participation if other family members have T1D or type 2 diabetes (T2D), despite the fact that T1D^5^ and T2D^6^ can have a familial component. As a result, we know little about how families manage T1D when multiple family members (MFM) are affected.

MFM with T1D may provide many resources. First, families may be more likely to recognize early symptoms of T1D, resulting in earlier diagnosis. Avoidance of diabetes‐related ketoacidosis (DKA) is associated with glycaemic levels that are more on target over time.^7^ Second, families may share knowledge about diabetes self‐care and technologies and may mutually support each other. Families with multiple generations affected by T2D communicate health behaviour information across generations, resulting in reciprocal support for self‐care behaviours.^8^ Third, individuals may engage in active disclosure among family members with T1D. In studies where one person in a family has T1D, disclosure to parents is related to better self‐care for adolescents and young adults.^9^ Disclosure to other family members who have diabetes may be especially beneficial for adolescents and young adults, as they report it is easier to disclose to peers who have T1D than those who do not.^10^ Finally, family members may engage in fewer attempts at obstructing self‐care behaviours, as they possess greater understanding of these behaviours. In a study where largely one person in the family had T2D, such harmful support was associated with higher HbA1c and lower self‐care.^11^

The literature on family members (without diabetes) living with a family member with diabetes indicates that family members experience challenges, ones that likely are compounded when MFM have diabetes. Siblings living with a sibling with T1D report stress over diabetes management, fears of ‘catching’ or inheriting the illness and anger regarding the disruption in their own activities (especially food‐related options and activities).^12^, ^13^ Siblings may incur more responsibility for siblings with T1D, creating distress and a feeling of exclusion that is detrimental for their own development.^14^ Parents caring for a child with T1D report parenting stress associated with medical care and financial burden,^15^, ^16^ which may be increased when MFM have T1D. Financial stress may be a particular concern for families with MFM with T1D as out‐of‐pocket expenses are substantial^17^, ^18^ and will likely accumulate with additional family members with diabetes. Mothers frequently report greater distress than fathers,^19^ potentially due to their greater involvement in the daily self‐care tasks of their children compared to fathers^20^ and such distress may be enhanced when multiple family members have diabetes.

For the person with diabetes (PWD), having MFM with T1D may mean that they are not only involved in their own self‐care but also in another family member's care. MFMs may be compared against one another, causing tension. Further, misinformation may also be shared across family members in how to manage diabetes with actual sharing of supplies (e.g., insulin, glucose strips) with potential benefits as well as liabilities associated with such sharing.^21^

In this study, we examined the lived experiences of families where MFM have T1D. The purpose was to: (1) explore how families with MFM with T1D use family history to shape early detection of symptoms and coping with T1D, (2) examine how families provide support to individuals with T1D and compare the support provided by members with and without T1D, and (3) understand how MFM with T1D may create a burden for some family members and the stressors that may come from having MFM affected. Detailed individual interviews with members of five families with MFMs with T1D were conducted, and grounded theory principles were used to code interviews to reveal themes capturing the lived experience of family members.

## 3 METHOD

### 3.1 Participants

In this single‐site, original study, families with two or more members with T1D in the nuclear family who were living together were recruited. Though we intended to recruit in the clinic, we started recruiting in March of 2020 and had to switch to using digital flyers on Twitter and T1D groups on Facebook due to COVID‐19. The target sample size was 10 families. However, we stopped at 5 families due to challenges in recruitment during COVID. Individuals were eligible if their family included two or more immediate family members diagnosed with T1D, were fluent in communicating and reading English, had access to a computer or smart device, and were at least 10 years of age. Participants 18 years of age and older were consented, and parental consent and individual assent were gathered for participants under age 18.

### 3.2 Data Collection

The university IRB committee approved study procedures. The research team included a psychologist, nurse practitioners, psychology post‐doctoral fellows, graduate students, and a study coordinator with previous experience in family and T1D research. Interviewers included two females and one male who were trained and followed a semi‐structured interview guide (Table 1). Interview guide questions were developed by the senior members of the team (MLL, CAB and NAM) based on the available literature on the effect of T1D on parents and siblings in families in which one family member had T1D and the collective experiences of the research team and the clinical experience of a team member who cared for MFM with T1D. Each interviewer had to pass off their skill set with two of the senior investigators. One interviewer experienced in pediatrics interviewed all of the children, while the other interviewers only interviewed parents. Coders were eight team members, working in two groups (MFM with T1D, family members without T1D).

**TABLE 1:** Examples of qualitative interview questions.

Family history	‘Was X (family member with T1D) diagnosed before or after you?’ (D) ‘How did you find out that they had T1D?’ (D, F)
Diabetes education	‘Where did you learn about diabetes and how it can be treated?’ (D, F) ‘Have you attended diabetes education courses?’ (D, F) ‘What do you think your risk is of developing T1D over the course of your life?’ (F)
Support receipt	‘Who of all these people (with T1D) would you put as the individuals who are your most important source of support? How about your next important source of support? Now who would you put as your least important source of support?’ (D)
Support provision	‘Do you provide help or support for anyone with T1D in your immediate family? If yes, choose the person you provide the most help to? What do you do to support the person? What are the positives and negatives of supporting this person?’ (D, F)
Family models	‘Which family member with T1D has had the most effect on how you manage diabetes?’ (D)
Family sharing	‘What sorts of things do you share with your family members about diabetes? Do you share your BG values, HbA1c?’ (D) ‘Have you ever shared supplies? Insulin? Advice about what pumps to use, CGMs, insurance programs?’ (D) ‘What sorts of things do family members share about diabetes? How do individuals share this information (in person, social media, apps, CGM share)? Who is it shared with?’ (F)
Disclosure	‘Who is your go‐to person when you need help and support for your diabetes?’ (D) ‘Do you ask X (family member) for support and how (in person, text)? What things are you willing to share with this person?’ (D)
Hindrance receipt	‘Who makes diabetes the hardest to manage? What kinds of things does the person do that make it hard?’ (D)
Hindrance provision	‘Do you do anything that could be perceived as unhelpful for X (family member with diabetes), even though you are trying to be helpful?’ (D) ‘Do you do anything that could be perceived as unhelpful for X (family member with T1D)?’ (F)
Diabetes & relationships	‘How do you think diabetes affects other members of your family?’ (D) ‘What would say are some of the positive/negative things that you have experienced because another member of your family has T1D?’ (D, F)

*Note*: D = persons with T1D; F = unaffected family members.

Data collection took place between March 2020 and June 2020. Five of seven families consented, with individual family members interviewed. Although whole families were recruited, any individual family member could decline to answer questions or withdraw from the study at any time. Saturation was reached after five families were enrolled and interviewed, at which point recruitment halted.

Interviews were conducted and recorded individually using HIPAA‐compliant video‐based conference software (Zoom) from the participant's home due to data collection being conducted during the COVID‐19 pandemic. Adult participants completed a pre‐interview survey including demographics, health and medical history along with questions about members in the family who had T1D and who were living in the household. We allowed participants to describe their experiences using a conversational structure. Each participant was asked to identify their ‘go‐to’ person and the person who provided them the most diabetes support. Interviewers and coders took field notes that were organized as observational, theoretical, methodological and personal.^22^ Interviews were audio‐recorded, transcribed verbatim and verified for accuracy.

### 3.3 Data Analysis

MFM with T1D is a relatively underexplored phenomenon where existing theories can only serve as a foundational base. Given the intersubjectivity of family life and our desire to understand how individual family members viewed their social world, a grounded theory approach was selected. Data were analysed using grounded theory principles and methodologies. Grounded theory is based on Symbolic Interactionism and focuses on people in a continuous process of giving shape to their worlds.^23^ As a research methodology,^24^ grounded theory generates theory from experiential data^25^, ^26^, ^27^ using systematic processes.

Using NVIVO version 11, investigators used a constant comparison approach^28^ to analyse the data^29^, ^30^ and explored similarities and differences in perceptions of diabetes management within and across families.^31^ Two independent investigators read the first two family interview transcripts to develop the initial codebook. Two coding teams then applied the codebook to family members with diabetes (MLL, MSC, MP and CM) and family members without diabetes (RGK, NAA, ELT and CAB). Open coding of successive interviews using a line‐by‐line analysis was used to apply data to existing codes or develop new codes. Matrices were developed to organize codes by family members with T1D (i.e., siblings and parent–child) and stratify by support (i.e., emotional and tangible).^32^ Field notes were triangulated in the analysis. Pedigree charts and comparative analytic tables were developed to understand family‐specific nuances for comparison.

## 4 RESULTS

### 4.1 Family Characteristics

Seven families contacted us and five families (*N* = 21) enrolled (see Table 2 for demographics). Family pedigrees (Supplement 1) highlight family members with and without T1D, the chronological order of T1D diagnosis, the ‘go‐to’ person in the family and the person with T1D identified as the most important source of support. The majority of family members nominated the mother without diabetes as the ‘go‐to’ person. In families 4 and 5, the father had T1D and was also nominated as the ‘go‐to’ person. In family 5, the child was too young to interview, but the mother and father both lived with T1D and indicated their spouse was their ‘go‐to’ person.

**TABLE 2:** Demographic information for parents and children (including adult children).

	All parents (*N* = 8)		All children (*N* = 13)	
	M (SD)	Range	M (SD)	Range
Age	38.9 (3.4)	34–43	13.8 (3.2)	10–20
Gender (% Women)	62.5%	—	53.8%	—
Race (% White)	87.5%	—	92.3%	—
Ethnicity (% Hispanic)	12.5%	—	30.8%	—
Educational level				
High school graduate	25.0%	—	—	—
Some college	12.5%	—	—	—
Associate degree	12.5%	—	—	—
Bachelors degree	37.5%	—	—	—
Masters degree	12.5%	—	—	—
Household income^a^				
Under $15,000	20.0%	—	—	—
$50,001–$60,000	20.0%	—	—	—
$90,001–$100,000	40.0%	—	—	—
More than $100,000	20.0%	—	—	—
Marital status (% married)	100%	—	—	—
Employment status				
Working full‐time	50.0%	—	—	—
Working part‐time	37.5%	—	—	—
Unemployed	12.5%	—	—	—
Health insurance (% yes)	100%	—	—	—
Self‐Pay Insurance (e.g., Health Exchange or Other Insurance)	20%			
Private Insurance	80%			

	Parents with T1D (*N* = 3)		Children with T1D (*N* = 9)	
Pump use (%)	0%	—	55.6%	—
CGM use (%)	100.0%	—	77.8%	—
Out of pocket monthly diabetes expenses				
$401–$500	66.7%	—		—
More than $500	33.3%	—		—

^a^ Household income is based on five households.

### 4.2 Themes

We first explore themes related to the benefits of having MFM with T1D for initial diagnosis and education, the role of the mother as a key source of support, the family cohesion and support that comes from MFM with T1D, and then the risks to family members that can occur.

#### 4.2.1 Theme 1: Initial Diagnosis

Family history of T1D was crucial to the early diagnosis of subsequent family members who exhibited T1D symptoms. Despite this early diagnosis, however, subsequent family members did not receive the same level of diabetes self‐management education and support (DSMES) because it was not offered by the clinic.

##### Family History is Crucial for Early Diagnosis

All five families had an extended family member with T1D (e.g. uncle, cousin and great‐aunt). This family history of T1D helped identify and diagnose T1D earlier in subsequent family members. For example, in family 1, the child was initially misdiagnosed with a viral syndrome, and it was not until the father recounted his own brother's diagnosis with T1D that T1D was considered. Initial glucose monitoring was done using the extended family member's glucose monitor, confirming elevated glucose. This child was subsequently diagnosed with T1D and hospitalized with DKA for 4 days. However, the other two siblings in the family who were subsequently diagnosed with T1D were identified right away. The mother reported, *‘After our first diagnosis … I was highly sensitive to what was going on with [my other children], just looking for signs [of T1D]’* (mother without T1D, family #1). In family 3, it was the maternal grandparents who recognized T1D symptoms from another grandchild with T1D. Earlier diagnosis meant hospitalization at diagnosis was avoided.

Four families (family 1, 2, 3 and 5) had undergone antibody testing (e.g. TrialNet) in other family members and knew there was a heightened risk of T1D in specific family members. This helped families be on alert for symptoms in those antibody‐positive family members. For example, in family 5, both parents with T1D found out about TrialNet through an online peer support community and had their son tested. He tested positive for T1D antibodies, resulting in early treatment.

##### Family Education

After the initial shock of being diagnosed with T1D, subsequent family members felt relieved that they already had some T1D knowledge. Family members described already knowing what to expect and what to do in certain situations. Formal DSMES was limited to the first child diagnosed within in families 1–4. *‘We just did mandatory [DSMES] for my first kid’* (mother without T1D, family #2). When subsequent family members were diagnosed with T1D, they relied on historical family knowledge to manage T1D and did not receive additional formal education. In family 5, the mother and father both lived with T1D; however, they received some formal DSMES specific to managing a toddler with T1D. Among families with siblings without diabetes, many siblings desired additional education for themselves so they could be helpful.

Despite the enhanced learning that existed when MFM have T1D, families relied on other sources for nuanced T1D information. Some mothers used diabetes online peer support groups. One mother received support from neighbours whose children had T1D, stating, ‘*It's helpful … just having somebody that you could get a hold of and say, “Hey, this happened to us. What do you guys usually do?”’* (mother without T1D, family #2). Similarly, another mother described, ‘*If there's a question that I have and my husband [who has T1D] is stumped, and I'm stumped, then I would go to [a diabetes online group] next’* (mother with T1D, family #5). One mother mentioned that she needed explicit assistance in struggling with the different aspects of having MFM with T1D, stating

When we had our second diagnosis, I think it would've been nice to know better how to juggle multiple diagnoses. And then also I would have liked more information on just like the financial and insurance aspects of it and how to make all of that work. (mother without T1D, family #3)

#### 4.2.2 Theme 2: Mother as Go‐To Person for the PWD: Mother is the Glue

Across all families, mothers provided the primary logistic, emotional and diabetes management support for family members. Mothers were labelled as the ‘go to’ person among all T1D family members interviewed and appeared to *be the glue* for the PWD. Mothers coordinated with school nurses, teachers and coaches to help manage diabetes during school and extracurricular activities, social functions (e.g., sleepovers), medical appointments and dealing with pharmacies and insurance companies. Mothers also juggled other health conditions such as coeliac and hypothyroidism.

##### Mothers' Increased Mental Load and Responsibilities

Mothers unanimously expressed feeling overwhelmed and in need of support. Fathers were rarely mentioned as providing substantial support by family members unless they had T1D themselves (families #4 and 5). Mothers bore the brunt of diabetes responsibilities, ‘*My husband has no clue what anything means. I mean, he knows diet. He knows more than maybe the normal person, but he really doesn't know anything because I know it’* (mother without T1D, family #2).

Mothers felt like they had to constantly remind their child, stating, *[The child] doesn't manage, then I pay for it at night because she'll decide to have a bedtime snack and not dose. I'm the one that deals with it … I cannot stand listening to myself, but if I don't ask, it doesn't happen. It's either, do I wait and just let her figure it out on her own and let her get sick so she takes it seriously, but then I don't want to chance doing permanent damage* (mother without T1D, family #1).

In addition to juggling the day‐to‐day needs of their families, mothers were also grappling with the future health of their families.

There's definitely an emotional part of it in a negative way. I have concerns for the future and what's going to happen with all of them. Also, since we know it's so prevalent in our family, I worry about our future generations, grandchildren. (mother without T1D, family #3)

Despite the increased mental load, some mothers described feeling grateful that they could assist their family.

Overall, I feel like the only negative is that it's weary on me emotionally. I get tired of it if I can be honest. I always feel like it's also a positive thing even though it wears me out. I'm grateful I can do that for them. And I do think they know that I'm there for them all the time, they know that I care. Even though sometimes I break down and I'm worn out, the positives are that we have a good relationship because they know they've had to rely on me and they can. (mother without T1D, family #4)

##### Mothers' Worsening Health

Mothers described a lack of sleep due to hypoglycaemia episodes and constant insulin pump or continuous glucose monitoring alarms. ‘*I will click on that app and see that they're all three within range, and it's shocking to me that I had slept for a 4‐hour stretch without having to get up’* (mother without T1D, family #1). Taking on these responsibilities impacted the mother's health.

We don't always get much sleep around here because of worry and stress and everything. After my daughter was diagnosed, I gained quite a bit of weight just from stress eating and not sleeping and not taking care of myself. And I haven't really ever gotten it off. It's been 5 years now and I'm still struggling to do that. (mother without T1D, family #3)

The added responsibility of managing MFM with T1D resulted in fewer opportunities for children to be left in the care of others (e.g., parent dates, child sleepovers). Mothers described feelings of constant worry, a weight on their shoulders and even feelings of a sense of loss.

I guess watching my kids struggle and not being able to fix it. Watching the highs and lows and seeing how much more responsible they have to be, and that they even have to think about that, planning emergency supplies. It takes some of their childhood away because they're just not quite as careless as I was as a kid. (mother without T1D, family #1)

#### 4.2.3 Theme 3: Family Support and Cohesion

Family members were viewed as experts in supporting those with T1D. While the support provided was overwhelmingly positive, there were some instances of negative support as well.

##### Families were supportive T1D experts

All family members were viewed as very knowledgeable about T1D, though expertise slightly differed given the developmental stage of the child and living situation. Family members without T1D often provided instrumental support (e.g., gathered rapid‐acting glucose to treat hypoglycaemia episodes, helped to count carbohydrates), ‘*We are all included with their diabetes by helping them with their carbs’* (sibling without T1D, family #1). Families also provided emotional support (words of encouragement, listening to someone vent). One sibling stated, ‘*I like supporting my sister. Whenever she feels down about having diabetes, I'm like, ‘hey you get to eat snacks all the time.’ I just try to make her feel better instead of making her feel bad’* (sibling without T1D, family #3). At times, family members without T1D struggled to balance how to be supportive without being controlling, or how to teach or guide instead of simply managing things for the PWD.

Sharing biophysical data was common across family members with T1D. Glucose levels were commonly announced at the dinner table prior to eating. For those families that used continuous glucose monitoring, data was shared across all family members, even siblings without T1D, who were old enough to have technology to follow the data electronically (e.g. app on mobile phone or iPad). Family discussions also included the latest research or technology in T1D.

##### Specific Support from Family Members with T1D

Participants with T1D identified and described family members with T1D as their most important sources of support (Supplement 1). This support was rooted in empathy, shared experience, role modelling and sharing tangible goods.

Difficult days were inevitable, and family members with T1D could deeply relate to another family member with T1D who might be struggling. One person described how their spouse with T1D understood how hard diabetes was: ‘*I don't have to spell it out all the time, [my husband] just gets it’* (mother with T1D, Family #5). Some family members with T1D also described how their family member with T1D ‘*understands a bit better on how to give support, and that sometimes you need cheering up’* (female sibling with T1D, Family #1).

The shared experience of T1D was extremely helpful. Family members were looked to as a sounding board to bounce off ideas and obtain advice without having to seek out the medical system. Shared experiences also provided unintentional reminders. For example, in Family #2, one sibling with T1D described seeing his sibling with T1D check their glucose and think, ‘*oh yeah, I need to do that too’* (male sibling with T1D, family #2). In Family 5, where every member had T1D, everyone checked their BG when they saw another family member checking their BG.

Role modelling was described by older family members as something they hoped to achieve for their younger siblings or children. Likewise, younger family members described looking up to older family members as being role models. One mother described role modelling with her two sons, stating, ‘*For one kid, it wasn't scary to give himself injections [when talking to his sibling about T1D]. That was good, like a role model kind of a thing. [Giving injections] wasn't a death sentence*’ (mother without T1D, family #2). Interestingly, those who were chronologically older but with less diabetes duration appreciated those with more diabetes experience. For example, the oldest child with T1D in family #1 indicated she looked up to her younger sister, who had lived with T1D longer, as a role model. Rarely, negative role modelling was present, ‘*Sometimes [my brother] doesn't take insulin and I was like, “Oh, maybe I don't have to.” And then I get super high, and it's just not good*’ (male sibling with T1D, family #2).

Tangible goods, such as insulin, glucose strips, pump supplies and continuous glucose monitoring sensors, were often shared among family members with T1D. Family #5 described putting all diabetes supplies in one drawer, and people could come and grab what they needed when they needed it. Family members with T1D often shared insulin in an emergency. One adolescent noted, ‘*Sometimes I do forget insulin on accident and then I realize it and I ask [my sibling] if I can use some of theirs for a little bit’* (male sibling with T1D, family #2).

##### T1D Strengthened Cohesion

Across family members, a heightened level of familial bonding was expressed due to T1D. Those with T1D could express empathy, bringing them closer, even among extended family members. One mother stated that a major benefit of having MFM with T1D is

Not having to explain everything to another individual. What to do, how to do it, some of the emotional factors that go along with it. It's just nice to have somebody that just gets it right away without having to try to make them understand. I think that's one of the hardest parts about communicating to someone else… (mother with T1D, family #5)

The husband concurred, stating ‘*Definitely we understand each other in kind of a way that, I mean, that's the extra level that most married couples don't have’* (father with T1D, family #5). One mother stated, ‘*They're closer with their uncle than I think they would have been otherwise. Especially when they're first diagnosed, they would call him and ask him questions’* (mother without T1D, family #1). Speaking about a niece with T1D, one father noted, *‘I feel like a positive has been that you form an extra bond. It was a family member [niece] that I don't feel like I was that close to before. And I feel like because of that we're closer to each other now’* (father with T1D, family #4).

Families mentioned that one of the benefits of having MFM with T1D was an increased vigilance on the overall health of the family. Families learned to carbohydrate count, made healthier food decisions and engaged in exercise. Families also worked to be inclusive. For example, Family #1 reported that they only have family treats if everyone can participate, which means that all three children with T1D must have their glucose in range to ensure that nobody feels left out.

In some instances, strong family support helped keep T1D in the background. A sibling with T1D stated, ‘*I go to my sister's soccer games, it's fun to see them just running on the field. When my sister feels a little low she'll do a little hand motion to my family. And then she'll run by and grab a glucose tab and keep running. It's fun to watch how you can still do whatever you need to without their diabetes getting in the way’* (female sibling with T1D, family #1). As families supported one another, they also described being more empathetic to other individuals and families with special needs.

#### 4.2.4 Theme 4: Family Stressors Among Family Members

Four challenges were described related to the financial cost of purchasing diabetes medications and supplies, frustrations among siblings without T1D, glucose interference with daily activities, and not respecting differences in management strategies.

##### Financial and Access Burden

The costs of diabetes were stressful across all families. One participant stated, ‘*My husband and I spend about 40% of our income on just medical care for type 1 diabetes’* (mother with T1D, family #5). Diabetes‐related financial burden weighed heavily on parents: *‘Two of my four kids have [T1D]. The hardest part is probably financially making sure that I have a good enough job that I can have insurance, that I can cover their needs’* (father with T1D, family #4). All three parents with T1D noted not being on insulin pumps due to financial concerns, though they were using CGM.

In addition to parents, children with and without T1D were concerned about how diabetes impacted current and future financial burdens. Financial burdens influenced how families saw the future of their members with regards to going to college, living independently, engaging in religious missions, and work opportunities. One mother stated, *‘I don't think it's going to be, ‘Hey, you turned 18 you're on your own. Figure out.’ I imagine that I'll be involved for as long as they'll let me. It's hard to even picture them not needing me ever. I'm sure financially we'll be helping them’* (mother without T1D, family #3).

##### Frustrations Among Siblings without T1D

Some siblings without T1D described that being in a family with multiple PWD had a negative impact on their emotional wellbeing. One sibling reported sadness and frustration that because of her father and siblings with T1D, ‘*we can't do as much stuff as other families’* (female sibling without T1D, family #4). In a few instances, siblings without T1D felt like they received less attention when compared to their siblings with T1D. In family #3, the sibling without T1D mentioned,

I feel really left out. I'm just so sad because I don't get to talk to my mom often because she's always worrying about [my siblings]. I talked to [my mom] about it and then she was like, “I know that you feel that way, but we really do need to pay attention to these kids because they have diabetes, this is a huge thing.” And so, I still feel a little bit left out. But I know that's how it has to be because they have to be taken care of. (female sibling without T1D, family #3)

When a subsequent sibling was taken to the doctor for suspected new onset T1D, the sibling without T1D went on to state, *‘it kind of felt my life just … crumbled a little bit, because then I knew that it [diabetes] was going to be a big part of our life and that she would feel left out and it made me kind of sad because I didn't want her to be sad’*.

From the parent perspective, some were concerned about how their children perceived the extra efforts they make for their child(ren) with T1D. One father stated, ‘*I sometimes worry that my oldest daughter resents things a little bit like, “Hey, how come I can't have a fruit snack?” And my wife will be like, “well, do you want diabetes? No. Okay.”’* (father with T1D, family #4).

Siblings without T1D felt like they needed to provide oversight when the mom wasn't available. One child stated,

At one point I felt like I had to be the mom, but then I talked to a counsellor because I also had huge anxiety issues…she was like, “You don't have to be the mom but you still need to watch over your sisters.” And so, I watched over them. But it's also hard when I'm trying to go and hang out with my friends and I have to take my sisters and watch over them. (female sibling without T1D, family #3)

##### Glucose Levels Interfering with Family Activities

Families reported a high incidence of T1D interfering with family activities involving being prepared for a variety of glucose scenarios. For example, one family described needing to pack diabetes supplies to address one family member's hypoglycaemia and another's hyperglycaemia during a hike. Difficulties surrounding preparation for trips (camping, vacations) were also described, as families had to consider access to hypoglycaemia supplies for multiple people, backup diabetes technology, and even destination temperatures for insulin storage. This increased effort was especially burdensome for parents.

Some participants reported that their ability to do certain activities as a family was *‘definitely’* dependent on the T1D management of *all* affected family members. One child noted,

If my blood sugar's high, none of us will get to eat ice cream or any sweets. We all have to have good blood sugars. Or when we go to [an amusement park] and … my blood sugar's low, we'll all have to wait for it to get up before we can go on a roller coaster (female sibling with T1D, family #1).

Although this strategy was reported to be positive and inclusive in some cases, at other times participants described disappointment and frustration with these family policies. Managing target glucose levels across several people can be difficult, and having to forego desired activities because of out‐of‐range glucose levels could be upsetting to family members.

In some instances, T1D interfered with parenting, which was especially challenging when both parents were experiencing hypoglycaemia at the same time. One father described, ‘*[My wife and I] both get low blood sugar at the same time. And our son's freaking out and trying to get us to do something, that can be kind of stressful’* (father with T1D, family #5). Whether dealing with similar or opposite glycemic excursions, glucose levels could influence a number of daily activities.

##### Differences in Diabetes Self‐Management Strategies

Competition and sibling rivalries were common. Across 4 of the 5 families, competition extended to glycemic levels and whether someone was putting enough effort into their T1D:

We kind of have competition, whoever has the best blood sugars throughout the day. Just helping each other out. Sometimes when me and my siblings get in fights, we'll be like, “Well, my blood sugars are better than yours today. You obviously don't even care.” And get in arguments about that. Or my sisters sometimes will be like, “Did you dose?” And I'll be like, “Yeah.” “You sure though?” And then after a while it gets annoying. They'll be asking 20 times. (female sibling with T1D, family #1)

Beyond competition, in some instances family members tried to help manage the insulin dosing of others, which had consequences. For example, in *family #1*, the oldest daughter (who has T1D) described grabbing her sister's insulin pump and dosing for her sibling, sometimes not realizing they had already dosed.

Individuals differed in how they managed T1D and how families responded to them. In some families, one child with T1D might be labelled the responsible one while another child might be labelled as less so. Occasionally, individuals with T1D would describe frustrations with other members with T1D who weren't doing as well with diabetes management or who managed their diabetes differently. Further, two people with T1D could eat the exact same meal and dose for the same amount of carbohydrates, and yet have different glucose responses.

Sometimes my brother does different things for himself, and I do different things for myself, but he then thinks he can critique me. Really, it's different for each of us, so that's a negative thing. They think they know what they know, but it's not really the same for everyone (male sibling with T1D, family #2).

Similarly, a mother with T1D stated, ‘*Even though my husband [who has T1D], understands, it's still a disease that manifests differently in everyone. Sometimes there are some hurdles with explaining how XYZ affects me versus him’* (mother with T1D, family #5).

## 5 DISCUSSION

The results reveal that T1D was a central feature organizing family life. Family members, both those with and without diabetes, were regularly involved in providing instrumental and emotional support. Generally, diabetes was thought to bring the family together with greater cohesion than if only one family member had T1D. Families were able to see the benefits T1D afforded the family in terms of the health of MFM and the closeness they were able to find in providing support to each other. This was despite numerous challenges mentioned by family members, including financial strain, disruption to daily activities, and stress, especially experienced by mothers.

A T1D diagnosis in one family member resulted in several benefits to the subsequent person with T1D. A T1D diagnosis in additional family members was made more quickly, with no one with a subsequent T1D diagnosis reporting DKA, consistent with large‐scale population‐based studies.^33^ Our results revealed that families used the symptoms and experience of the first family member diagnosed with diabetes as guides for diagnosis and guidance for diabetes management. Although the use of family history to guide diagnosis was generally positive, formal DSMES in subsequent family members was not offered. However, family members indicated they would have benefitted from additional education. A modified version of DSMES should be explored for MFM with T1D.

Numerous benefits to having MFM with T1D were noted, including expert advice from family members with T1D. In MFM with T1D, processes of disclosing diabetes information and family members soliciting that information were common and happened rather seamlessly in family life. Our prior work in families with one family member with T1D revealed that disclosure and solicitation of diabetes problems between a parent and an adolescent/young adult with T1D are beneficial for self‐care.^3^, ^9^ Therefore, it is likely that the greater frequency of disclosure and solicitation in families with multiple members with T1D is of benefit for self‐care. The importance of sharing the lived experience of T1D may be especially beneficial for such disclosure processes to occur among family members with T1D.^34^ Individuals with T1D may avoid engaging family members if they do not understand T1D.^35^

Several challenges were revealed in family members with MFM with T1D. Mothers, who were consistently the go‐to person of those with T1D, were experiencing substantial distress and disruptions in sleep that were affecting their own health. The fact that mothers reported greater distress than fathers is consistent with the literature on parenting stress in families where children have T1D.^19^ Clearly, mothers' sleep was disrupted, which likely has consequences for their health over time. Mothers need greater support, potentially from other family members, such as fathers or extended family members, friends, healthcare providers, and peers who support multiple individuals with T1D in their family. Stepped‐care interventions that provide tailored levels of support to mothers may be beneficial and reduce the caregiving burdens of mothers.^36^

Sibling support provided was not generally reported as burdensome. However, some siblings felt especially distressed by diabetes prevalence in the family. The extreme distress expressed by one female sibling suggests that families need to be mindful of the burden among siblings who may be asked to take on additional caregiving, bordering on ‘parenting’ roles in the family that are developmentally inappropriate and unsafe.^37^ This increased responsibility of siblings to take on the role of the parent (in the extreme referred to as parentification) has both harmful as well as positive effects on sibling relationships and well‐being in the broader developmental literature.^38^ In the case of T1D self‐care behaviors, making sure that siblings have the necessary education so that self‐care is safe is important.

The majority of participants reported that the financial burden of diabetes was a significant source of stress. The financial impact of diabetes has been well described,^17^, ^21^, ^39^, ^40^ though this is the first paper to describe the struggles families face when MFM have T1D. The pervasiveness of the concern mentioned not only by parents but also by children (those with diabetes and without) suggests the need for additional support for these families. Financial toxicity occurs through direct expenses, indirect costs, insurance coverage limitations and structural access barriers as identified by Patel,^41^ which were identified by participants. Helpful resources, such as the T1D financial toolkit,^42^ are underway and could be expanded in the future for those families who manage MFM with T1D. To manage T1D is to increase a family's risk for financial toxicity, which only compounds with each additional family member with T1D. Health policies are needed to protect families impacted by T1D from financial toxicity by implementing regulatory cost controls with pharmaceutical and device companies, mandating transparent price structures and expanding coverage for essential medications and diabetes technologies.

This study should be interpreted in the context of limitations. First, perceptions of family members and their own experiences were relied on. Observing family members in their natural environment may have yielded additional rich data about family processes.^43^ Such observations may have given a more nuanced view of the involvement of some family members, especially of fathers. However, our team was not able to conduct home interviews due to COVID‐19 restrictions. Second, the study involved a small number of families, including four traditional and one blended family, and included both children and parents with T1D. However, most participants in this study were non‐Hispanic White. Perspectives from ethnically and racially diverse families may have yielded different family dynamics and social supports, especially since intergenerational living is more common among diverse groups (Campos & Kim, 2017). Finally, given that our focus was on MFM with T1D, we cannot compare findings to families where only one person (parent or child) is living with T1D, though some interventions may be generalizable. Individual or family counselling by clinical psychologists specializing in T1D, support groups, and, as noted above, a modified DMSES programme for MFM with T1D. Diabetes camp, especially one designed for MFM with T1D, may be helpful in providing support to all members of the family, including support for mothers with caregiving burdens, siblings without diabetes that may need additional attention and perhaps even mobilizing fathers to be more engaged with T1D caregiving. This is an opportunity for future work to identify when more novel interventions are needed based on family dynamics.

### 5.1 Clinical Implications

Several family members who were diagnosed later than other family members indicated that they would have benefitted from their own education regarding T1D, rather than relying on the education that parents or older siblings might have received. Endocrinology providers and diabetes care and education specialists need to be attuned to providing DSMES to subsequent family members with T1D in addition to tips for managing MFM with T1D. Family trips were frustrating and clinicians can help prepare families on how to manage MFM on vacation. In addition, mothers may especially benefit from support services that deal with juggling the added responsibilities of MFM with T1D. These families experience high financial burdens due to the exponential cost of having MFM with T1D and resources on how to optimize insurance coverage and manage finances are needed. Identifying strategies for siblings without T1D, who are stressed, though likely not attending clinical appointments, is needed to address whole family care. Lastly, while it is recommended that people with diabetes receive DSMES at diagnosis, at times of transition, with any complicating factors, and annually,^44^ it may be beneficial to assess whether and how often family members need DSMES as well.

## FUNDING INFORMATION

Funding was provided by the University of Utah's Driving Out Diabetes, a Larry H. Miller Family Wellness Initiative.

## CONFLICT OF INTEREST STATEMENT

MLL is on the Professional Advisory Board for DiabetesWise and a consultant for dQ&A; both are unrelated to this study.

## Supporting information


Data S1.
