# Supplementary material for: Family member involvement when multiple family members have type 1 diabetes
Source: Diabet Med. 2025 Oct 6;42(12):e70145. doi: 10.1111/dme.70145 (PMC12628722; doi:10.1111/dme.70145)
Supplement: Supplementary file 1 — Data S1. [file DME-42-e70145-s001.pdf]

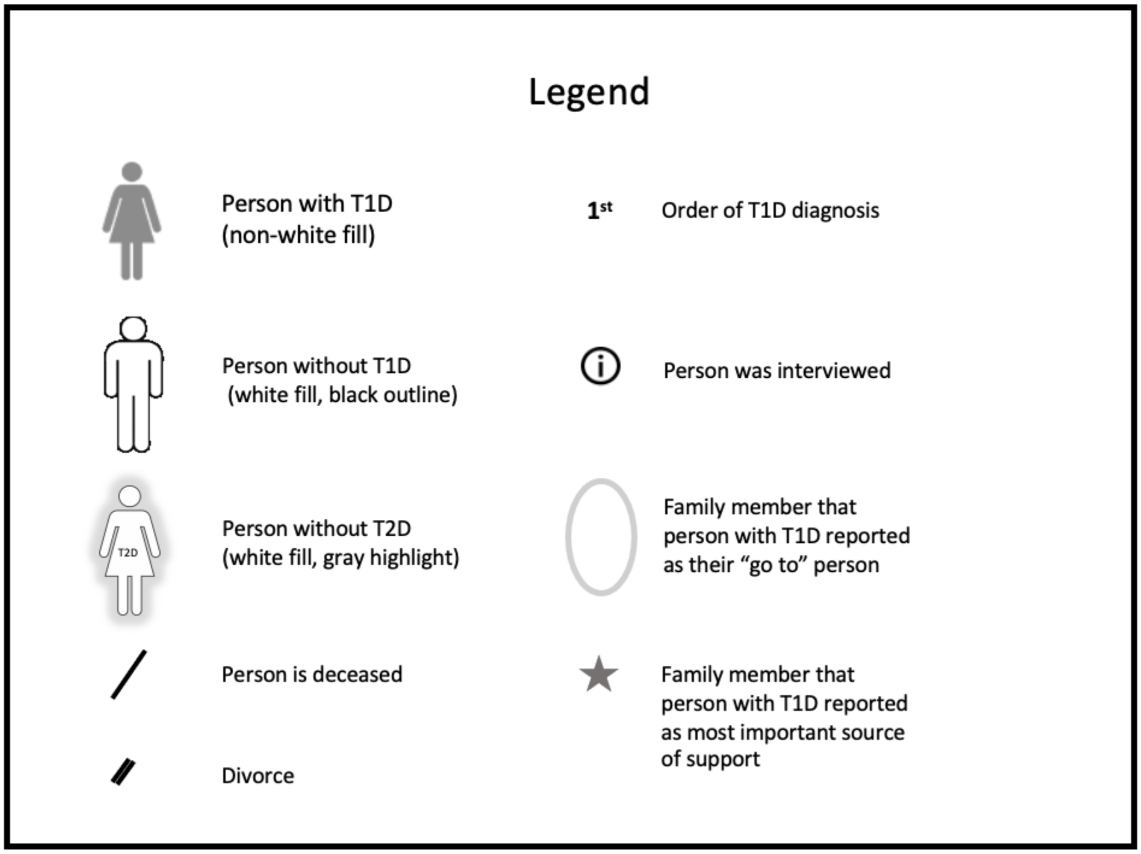
Legend
Person with T1D
(non-white fill)
1st
Order of T1D diagnosis
Person without T1D
(white fill, black outline)
Person was interviewed
T2D
Person without T2D
(white fill, gray highlight)
Family member that
person with T1D reported
as their "go to" person
Person is deceased
Family member that
person with T1D reported
as most important source
of support
Divorce

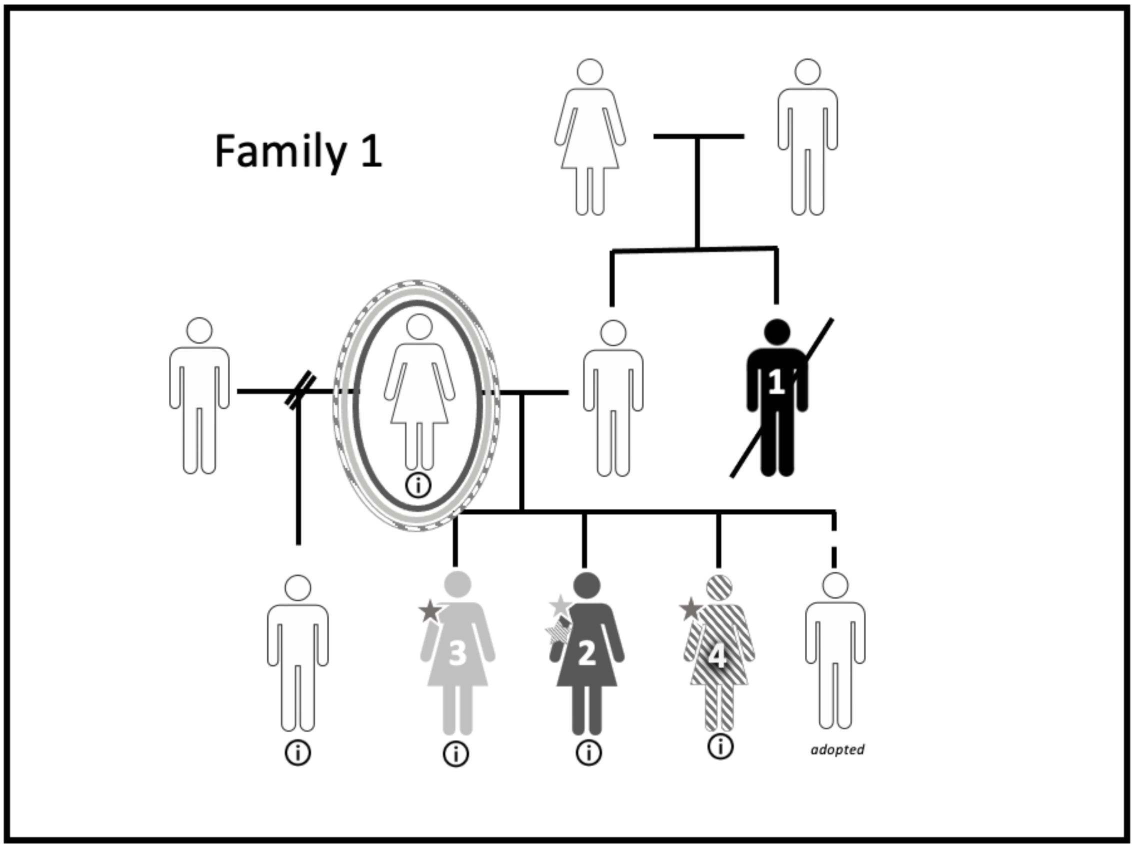
Family 1
1
3
2
4
adopted

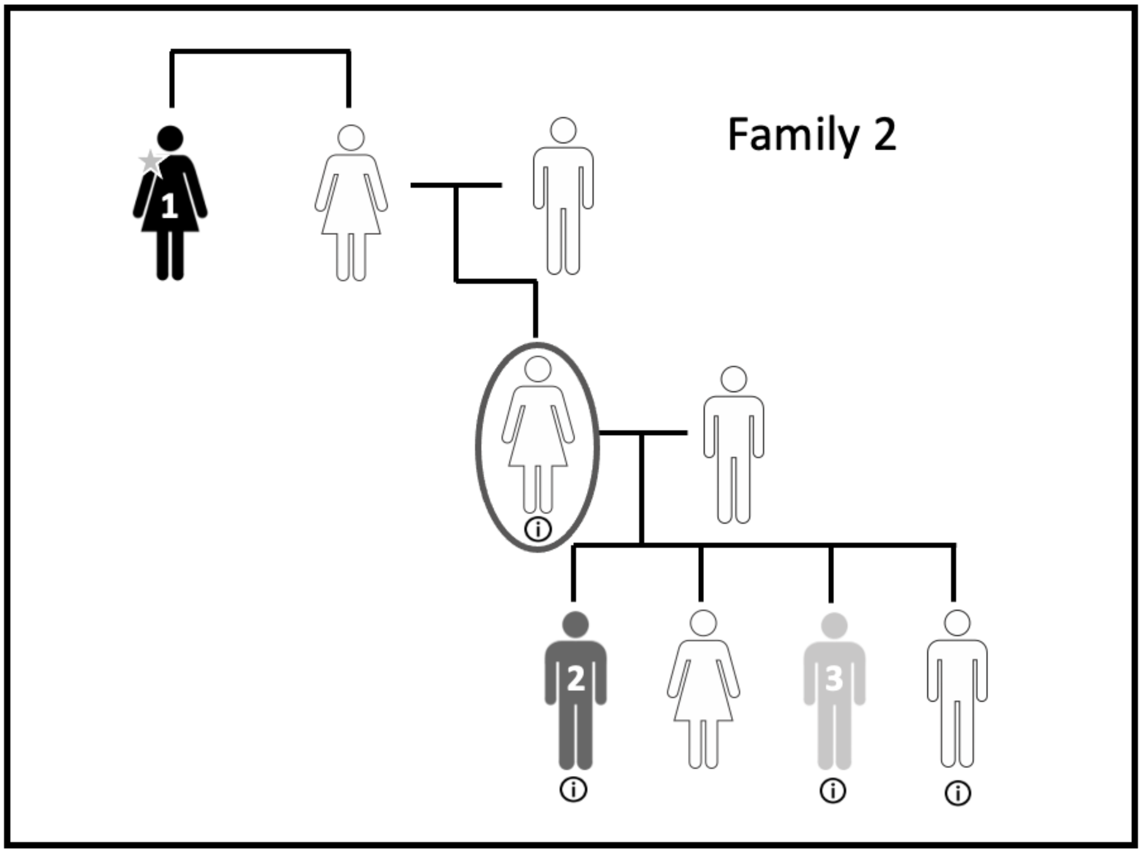
Family 2
1
2
3

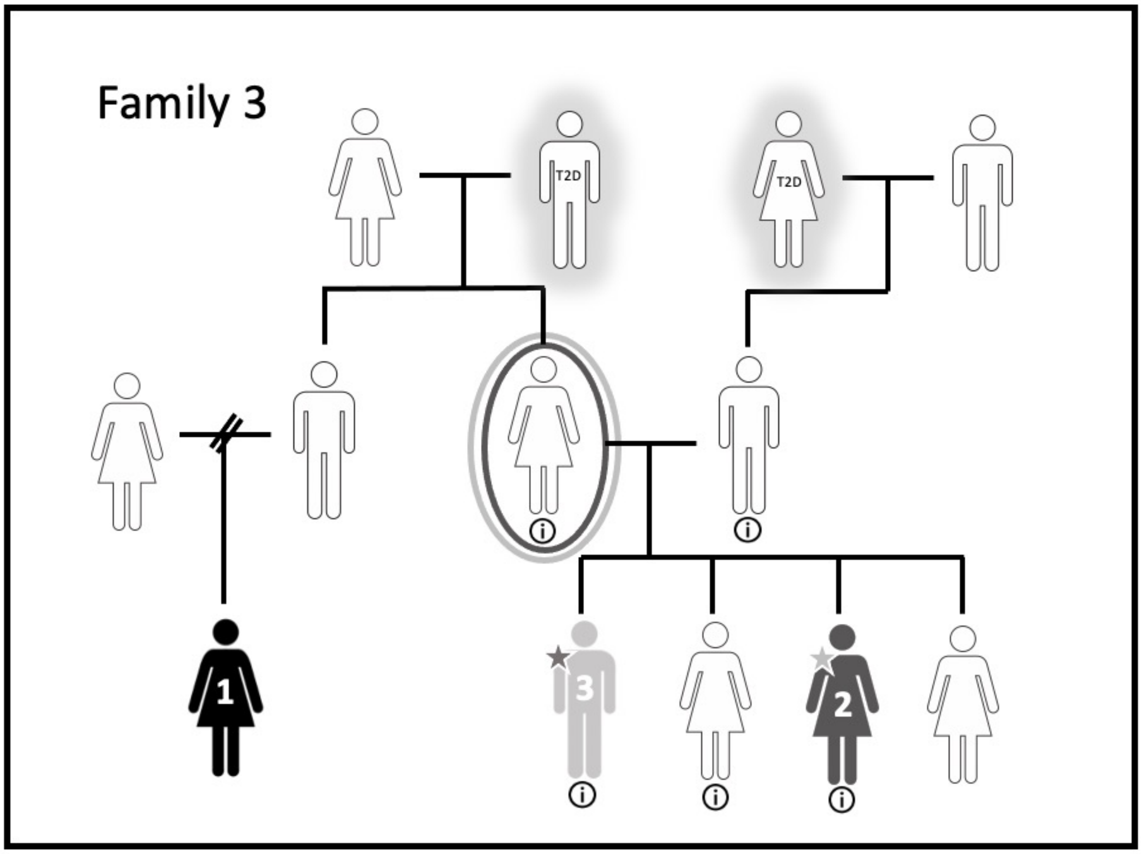
Family 3
T2D
T2D
1
3
2

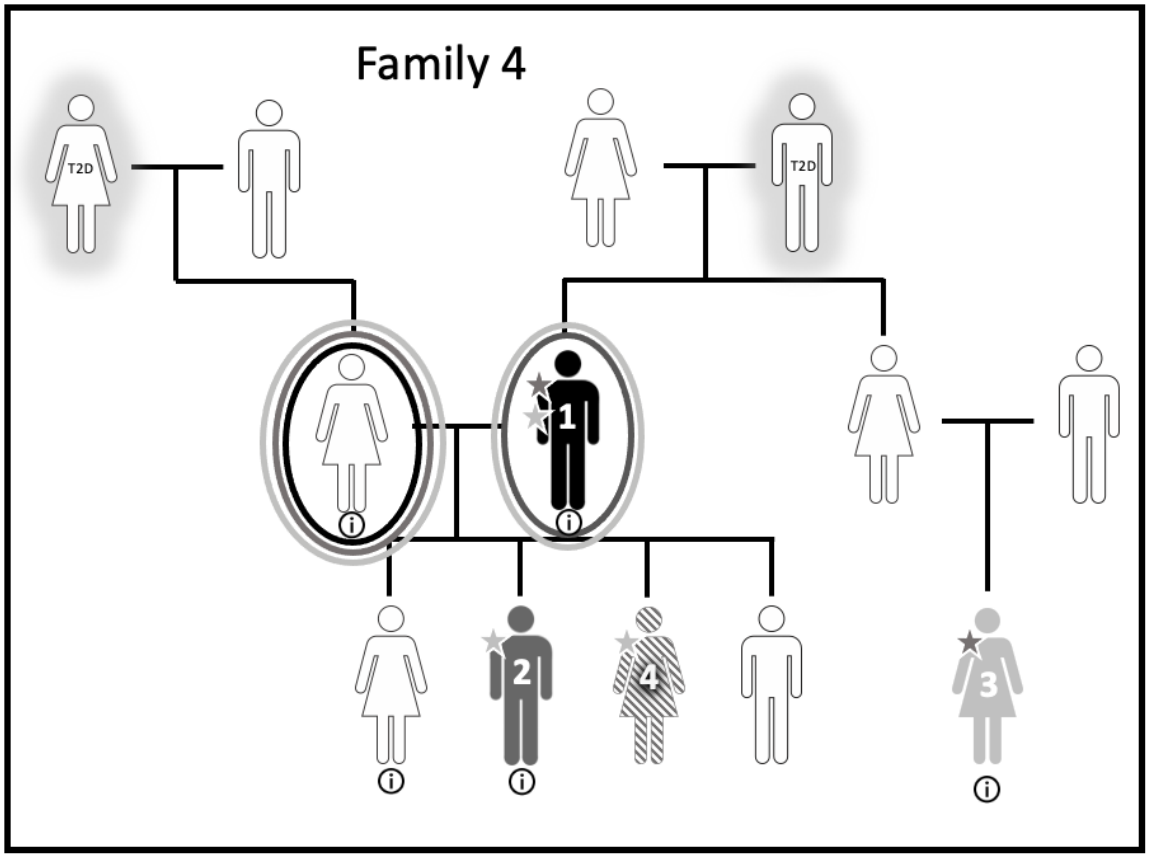

Family 4
T2D
T2D
1
2
4
3

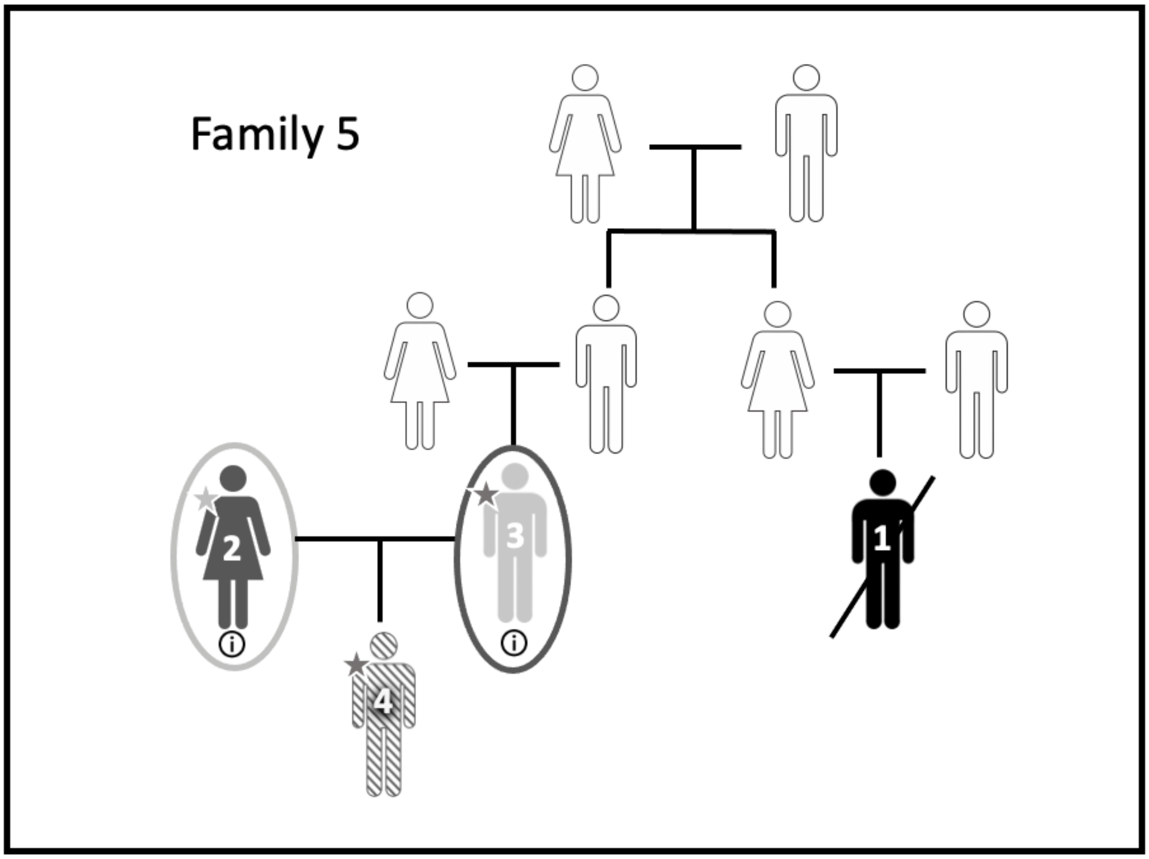

Family 5
2
3
1
4
